# Psychological and Psychopathological Aspects of Kidney Transplantation: A Systematic Review

**DOI:** 10.3389/fpsyt.2020.00106

**Published:** 2020-03-05

**Authors:** Concetta De Pasquale, Maria Luisa Pistorio, Massimiliano Veroux, Luisa Indelicato, Gabriella Biffa, Nunzialinda Bennardi, Pietro Zoncheddu, Valentina Martinelli, Alessia Giaquinta, Pierfrancesco Veroux

**Affiliations:** ^1^Department of Educational Sciences, University of Catania, Catania, Italy; ^2^Vascular Surgery and Organ Transplant Unit, University Hospital of Catania, Catania, Italy; ^3^SIPsiTO, Italian Society of Psychology and Psychiatry of Organ Transplants, Catania, Italy; ^4^Department of General Surgery and Medical-Surgical Specialties, University of Catania, Catania, Italy; ^5^Department of Surgery, Transplantation and Advanced Technologies GF Ingrassia, University of Catania, Catania, Italy; ^6^Clinical Psychology and Psychotherapy Unit, San Martino Hospital—Genoa, Genoa, Italy; ^7^University Hospital, City of Health and Science, Turin, Italy; ^8^Department of Mental Health, Bergamo Local Health Authority, Bergamo, Italy; ^9^Department of Brain and Behavioural Sciences, University of Pavia, Pavia, Italy

**Keywords:** psychopathology, depression, anxiety, cognitive disorders, sleep disorders, adherence, social functioning, kidney transplantation

## Abstract

Kidney transplantation is a serious event that involves profound psychological, relational and social changes both for the patient and his family context. Assessment of personality profile, awareness of disease, family and social support of the patient candidate for kidney transplantation are necessary because factors not adequately considered, can influence the success of the transplant and alter the psychological stability of the patient. The present study aims to provide a systematic review of the literature of the last twelve years (2006–2018), focusing in particular on patient’s readiness level and illness management and on possible psychopathology. Sixty-two studies were examined. Based on the Downs and Black checklist, most studies (n = 32) were of high quality; 15 of which related to lifestyle, health education, and therapeutic adherence in post-renal transplantation, 17 studies concerned the possible existence of psychopathology and cognitive impairment of renal deceased transplanted subjects. The literature used has shown that the population of kidney transplant patients is exposed to a high risk of psychiatric disorders with repercussions on the quality of life and the risk of rejection. Therefore, an adequate pre-transplant psychosocial assessment is necessary, which allows a more in-depth knowledge of the candidate to plan coping strategies and possible post-transplant psychotherapy.

## Introduction

Kidney transplantation is a valid treatment option for end-stage renal disease, the only one capable of correcting, in addition to the emuntory function, also metabolic, hematological and endocrine abnormalities, allowing most patients to obtain a better quality of life (1–6).

Despite the progress of medical science and technology in this field, remain problems that affect the amount of transplants implemented as well as their success. In addition to the insufficient number of organs donated by deceased or living donors, one of the main difficulties is the management of the pre- and post-transplant pathways, often exclusively medical-surgical, which excludes the importance of integrity between mind and body. Transplantation is a very demanding and particularly stressful event that requires the patient to implement his bio-psycho-social skills in order to accept and integrate the new organ physically and mentally. This surgery, therefore, involves numerous psychological, existential, affective, relational, and social changes both for the patient candidate and for his family context. (7–9).

In transplantation, surgery allows rapid functioning from the anatomical and physiological point of view, but cognitive and emotional integration is also necessary (“psychological transplant”) (10–12).

Surgery such as transplantation is a time of great stress that threatens the sense of continuity and personal integrity, causing strong emotions and can alter one's personal identity, with the possible onset of psychopathology and psychosocial problems (10, 13–15).

The psychopathology or insufficient internal and external resources of the subject can lead to a poor adherence to post-transplant pharmacological treatment, greater probability of rejection, and greater probability of beginning of organic pathology (16, 17).

The term adherence refers to the concept of continuity to medical prescription (drugs, diet, lifestyle) and patient satisfaction. In this perspective, adherence is understood as a sharing based on the therapeutic “alliance” between doctor and patient respecting the needs of both. The term compliance, on the other hand, represents the patient's obligation to follow drug therapy and therefore assumes a negative connotation (18).

In this context, it is necessary to evaluate the psychological profile and the personality of the patient who is a candidate for kidney transplantation in order to prevent factors that are not adequately monitored from influencing the positive outcome of the surgery or causing emotional disorders for the subject himself (15–21). At the same time, the evaluation of the family and the social context, of which the patient is a part, play a fundamental role. This allows us to investigate whether the family environment is favorable in terms of social, material and emotional support for the candidate, and through the analysis of communication between the various family members, it allows us to understand the fears of the path undertaken both in the pre that in the post intervention (22, 23).

Recent literature shows that patients who receive psychotherapy support in the pre- and post-transplant areas improve treatment compliance and limit possible anxiety and depression. Patients seem to recover a significant quality of life with changes in physical aspects but to an even greater extent in emotional and psychological ones (4, 22).

Regarding the close relationship between psychopathology and therapeutic adherence, De Pasquale et al. (24), examining the literature, analyzed the psychopathological aspects in kidney transplantation, affirming that the presence of a multidisciplinary team allows the transplanted patient greater adherence to therapy, the use of new coping strategies and the adoption of more appropriate lifestyles.

The aim of the present paper is to offer a systematic review of the literature of the last twelve years (2006-2018), on the psychopathological and psychological aspects and on awareness disease of adult kidney transplant subjects. Since the psychopathological complications, if present in the kidney transplanted subject, represent a risk factor for non-adherence to the treatment, the authors intend to analyze whether a correct psychological, psychiatric evaluation in pre and post transplantation and patient support interventions for the disease management, can improve long-term transplantation results, avoiding non-adherent behaviors and negative outcomes such as rejection. A well-structured assessment which includes information regarding the personality, possible psychopathology, the experience of illness and the motivation to transplant, could thus be used as a basis to guide experts in the sector towards a homogeneous clinical activity, and identifying most appropriate treatment for a “best practice” (25).

## Methods

### Data Source and Selection Criteria

A literature search has been performed following the main research items reported in literature during last 12 years. The literature was examined using the preferred reporting items for systematic reviews and meta-analysis (PRISMA), a checklist of 27 items based on the Cochrane Consumers and Communication Review model (26).

The following keywords have been selected for research: “(self-efficacy, coping, health education, anxiety, depression, psychopathology, awareness diseases, social support, family support, quality of life, body image, adherence, compliance, psychosis, personality disorders, sleep disorders, neuropsychological disease) and (kidney transplant).”

For the research we used databases such as: MEDLINE, Scopus, Embase, PsycINFO and Cochrane Library which combine free terms and controlled terms (MeSH: Medical Subject Headings).

The topics chosen to deepen the study referred to the 4 domains of the Stanford Integrated Psychosocial Assessment for Transplantation (SIPAT) (27) that is: 1. “Patient’s readiness level and illness management;” 2. “Social support system level of readiness;” 3. “Psychological stability and psychopathology;” 4. “Lifestyle and effect of substance use.” SIPAT is a reliable tool that allows us to predict the psychosocial outcome of transplantation (positive or negative), its strength is the standardization of its evaluation process (27).

Only full/text manuscript concerning clinical studies on adult (>18 years) deceased donor kidney transplantation published in peer-reviewed journals in English between January 2006 and December 2018, were included in the selection.

The reference lists of the articles included in the review have been checked manually by several independent authors (CDP, MLP, LI, MV) for the selection of the most relevant studies. Moreover, members of the Board of Directors of the Italian Society on the psychological and psychiatric aspects of organ transplants (SIPSITO) were also contacted as experts in the field (PZ, LB, GB).

**Figure 1:**
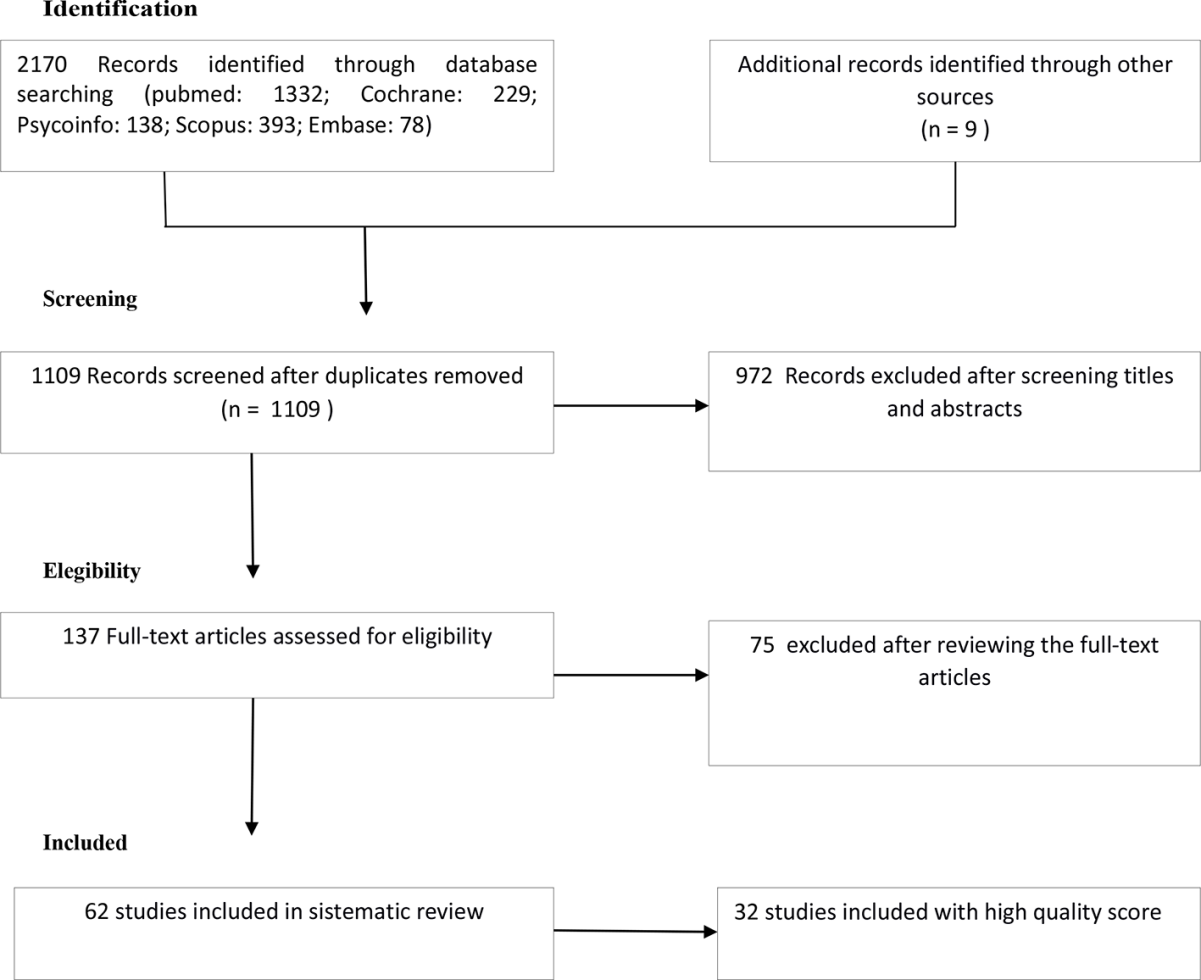
Flow diagram of inclusion procedure.

Exclusion criteria included non-English documents, studies that included adolescents or children (age < 18 years) and articles with no abstract or incomplete results. **Figure 1** summarizes the research strategy used to select the studies (identification, screening, eligibility, inclusion process) in the critical review.

#### Recorded Variables

The variables recorded for each article included in the review were: author (s), year of publication, country, keywords, study design, title, sample (age, gender), measurements, results (**Tables 1** and **2**), quality score (**Table 3**).

**Table 1:** High quality studies (≥19).

	First author, years, country	Publication year	Key words	Study design	Title	Sample	Measure	Results
1	Burkhalter et al. (28) Switzerland	2013	Renal transplantation, Sleep disturbances, Sleep quality, Daytime sleepiness	Cross- sectional	Self-reported sleep disturbances in renal transplant recipients.	249 kidney transplant (KT) adults; Mean age 59,6 years	PSQI, SOS	The most frequent sleep problem was difficulty staying asleep (49.4%), followed by problems falling asleep (32.1%). The most prevalent sleep disturbance was the need to urinate (62.9%) and reduced daytime functionality (27%).
2	Burkhalter et al. (29) Switzerland	2015	bright light therapy; randomized controlled trial; renal transplantation	Randomized controlled trial	The effect of bright light therapy on sleep and circadian rhythms in renal transplant recipients: a pilot randomized, multicenter wait-list controlled trial.	30 home-dwelling KT patients randomly assigned 1:1 to either 3 weeks of BLT or a wait-list control group.	Wrist actimetry (measuring sleep and circadian rhythms), DASS 21, SCWT and melatonin assay (circadian timing) were used.	The bright light therapy (BLT) improved significantly sleep timing, while had no significant effect on circadian and sleep measures. BLT improved depressive symptomatology in the intervention group (baseline-intervention: 5.92–5.75 [SE: -0.28 (-0.87; 0.31)] and from intervention to follow-up: 5.75–4.08 (score >5 means depressive symptomatology), [SE: -0.52 (-1.12; 0.08)]; cognitive executive function (i.e. Stroop test results) did not change.
3	Corruble et al. (30) Paris	2011	depressive symptoms, self-assessment, transplantation, liver, kidney, outcome	Longitudinal Prospective	Report of depressive symptoms on waiting list and mortality after liver and kidney transplantation: a prospective cohort study	339 KT adults Mean age 48 years	BDI-Short Form, STAI	51.6% of patients reported depressive symptoms on waiting list, 16.5% had a graft failure. No significant effect of depressive symptom intensity was shown on 18-month graft survival. 6.67% of patients had a graft failure among the 90 individuals with a Short-BDI score higher than 7 at baseline. The study shows a better 18-month post-transplantation outcome for patients who had a psychiatric history of depression before transplantation.
4	Cukor et al. (31) USA	2009	depression, Hemodialysis, Adherence, kidney transplantation,	Longitudinal Observational	Depression is an important contributor to low medication adherence in hemodialyzed patients and transplant recipients.	159 adults: 94 KT recipients and 65 patients in hemodialysis mean age 46,9 years	BDI, MTAS	Compared to the transplant group, the hemodialysis cohort was significantly more depressed. They also had a significantly lower adherence to medication. Depression was the only statistically significant predictor of medication adherence beyond gender and mode of treatment.
5	De Pasquale (32) Italy	2014	Self-efficacy, kidney transplantation, quality of life	Cross-sectional	Role of “self-efficacy” in the process of long term care in kidney transplant recipients	120 KT adults Mean age 40,5 years	GSE, SF-36, SCL 90 R	Self-efficacy is positively correlated with both physical role limitations and mental health. With increasing self-efficacy there was a decrease of psychic symptoms as investigated with the use of the SCL90 R test
6	De Pasquale et al. (16) Italy	2016	Transplantation; Adherence; Mental health; Psychological assessment; Psychiatric assessment	Observational	Psychological perspective of medication adherence in transplantation	74 KT adults mean age of 48.3 years	SCL-90 R TEMPS-A, PSDA, SF-36, BAASIS	Individuals with a higher level of education and more years since transplantation showed better mental balance. Regarding gender, women appeared to be less adherent to therapy. Further, the years since transplantation adversely affected the proper pharmacological assumption. Adherence to therapy did not significantly change with the mental health index
7	De Pasquale et al. (9) Italy	2010	Mental rigidity, egocentrism, hypercontrol, Body image, kidney transplantation, Emotional coarctation	Cross-sectional	Body image in kidney transplantation.	20 KT adults, mean age 39.5 years	DAP	“Emotional coarctation” (V1) in the sense of “mental rigidity,” “egocentrism,” and “hypercontrol” were present in all transplant recipients (100%); “Difficulty in interpersonal relationships” (V3) and “anxiety” (V5) were present in 70% of transplantation patients.
8	Denhaerynck (33) Switzerland	2007	Non adherence, Immunosuppressants, kidney transplant, self-efficacy	Longitudinal Prospective	Prevalence and risk factors of non-adherence with immunosuppressive medication in kidney transplant patients.	249 adults Male 141 (56.6%) aged 18 and over	EM	Non adherence was associated with lower self-efficacy. Higher self-reported non-adherence were associated to no pillbox usage, and male gender. Adherence declined between Monday and Sunday.
9	Fructuoso et al. (34) Portugal	2011	kidney transplant, Hemodialysis, Peritoneal Dialysis, Quality of life, chronic kidney disease	Randomized controlled trial	Quality of life in chronic kidney disease.	60 adults: 30 with chronic kidney disease (CKD), Male 43.3%, mean age 61,2; and 30 with Kidney Transplant (KT), Male 50%, mean age 51,8	SF36, KDQOLSF	All patients with (CKD) presented better results in the “Social Functioning “ scale and the lowest results appeared in the “General Health” scale. The data showed a general improvement in quality of life after transplantation compared to dialysis patients. “Health” scale was better results in peritoneal dialysis patients comparing to hemodialysis patients. Age, gender and hemoglobin level interfered with “general health” in all patients.
10	Gelb et al. (35) Canada	2008	Anxiety; chronic kidney disease; depression; kidney transplant; neuropsychological	Cross- sectional	Cognitive outcome following kidney transplantation	136 adults, 42 KT recipients, 45 outpatients with pre -dialysis and 49 healthy controls, mean age 59,67	CVLTII, D-KEFS, IADL	Findings indicated that TX and CKD patients demonstrated significantly worse verbal learning and memory in comparison to controls. CKD patients performed significantly worse on a set-shifting task, than TX.
11	Gentile et al. (36) France	2013	Associated factors, Cross-sectional multicenter study, Quality of life, Renal transplant recipient,	Cross- sectional	Factors associated with health-related quality of life in renal transplant recipients: results of a national survey in France.	1061 KT adults, aged 18 and over	HRQOl, SF36	The variables which decreased a good QOL were: females, unemployment, lower education, living alone, high BMI, diabetes, recent critical illness and hospitalization, non-compliance, a long duration of dialysis and treatment side effects. The most predictors of worse QOL were: side effects, infectious disease, recent hospitalization and female gender.
12	Gheith (37) Egypt	2008	Kidney transplantation, Compliance sexual activity die, cancer, prevention	Longitudinal Observational	Compliance with recommended life style behaviors in kidney transplant recipients: does it matter in living donor kidney transplant?	100 KT adults, Aged 18 and over	Surveys on compliance with the immunosuppressant therapy and with recommended lifestyle behaviors.	Most of the kidney recipients were compliant with the immunosuppressants. The women were less compliant than men with medications (P = .02), and poor compliance with medications was more frequent among those with living unrelated donors (P = .04). Kidney transplant patients had good compliance with immunosuppressive medications, but not with most of the recommended behaviors.
13	Goedendorp (38) The Netherlands	2013	creatinine, fatigue, functional impairments, kidney transplant recipients, proteinuria, psychosocial factors.	Longitudinal panel	Severe fatigue after kidney transplantation: a highly prevalent, disabling and multifactorial symptom.	180 KT adults, aged 18 and over	CIS, SIP, SF- 36, BDI-Primary Care, SLL- I/D	KT recipients were significantly more often severely fatigued (39%) compared to matched population-based controls (22%; P = 0.001). Severe fatigue after kidney transplantation is more strongly related to behavioral and psychosocial factors than specific transplantation related factors.
14	Gordon et al. (39) USA	2010	kidney transplant self-efficacy, adherence, physical activity, social functioning	Cross-sectional	Prevalence and determinants of physical activity and fluid intake in kidney transplant recipients.	88 KT adults aged 18 and over	PASE, SSS, SF36	76% of patients were sedentary with a quarter exercising either regularly (11%) or not at current recommendations (13%). One third (35%) reported drinking the recommended 3 L of fluid daily. Multivariate analyses indicated that private insurance, high self-efficacy, and better physical functioning were significantly associated with engaging in physical activity (p < 0.05); male gender, private insurance, high self-efficacy, not were oneself responsible for transplant success but were significant predictors of adherence to fluid intake (p < 0.05). Younger age, high value of exercise, and higher social functioning significantly (p < 0.05) predicted high self-efficacy for physical activity, while being married significantly (p < 0.05) predicted high self-efficacy for fluid intake.
15	Gross et al. (40) USA	2010	Mindfulness, stress, transplant, kidney, waitlist, quality of life, sleep, anxiety, depression	Randomized controlled trial	Mindfulness based stress reduction for solid organ transplant recipients: a randomized controlled trial.	137 adults: 71 KT, 66 on waitlist, mean age 54 years	STAI, CES-D PSQI	MBSR reduced anxiety, sleep symptoms (P < .02) and depression (P < .01)
16	Kofman et al. (41) France	2018	Bipolar disorder; kidney transplantation; psychiatric outcome; psychosis; transplant outcome	retrospective multicenter cohort study	Safety of renal transplantation in patients with bipolar or psychotic disorders: a retrospective study	Forty-seven KT recipients including 25 women were identified, 34 with BD and 13 with psychotic disorder.	Psychiatric assessment regarding pre- and post-transplant treatments, need for new drugs or new approaches such as electroconvulsive therapy, psychiatric relapse (PR), hospitalization, discontinuation of treatment, and suicidal attempt.	Patients' overall cumulative death rates at 60 months were not significantly different in both groups [12.2%; 95% confidence interval: (4.5-24.1) in the group with psychiatric disorder versus 5.2%; (1.7-11.7) in control group P = 0.11]. Twenty-three patients (16 with BD and seven with psychotic disorder) experienced at least one psychiatric relapse [incidence rate: 1.8/100 persons- months; 95% CI; (1.2-2.7)] totaling 13 hospitalizations within 60 months of follow-up. Four patients stopped immunosuppressive therapy leading to allograft loss in three.
17	Kovacs et al. (42) Hungary	2011	quality of life, fatigue, waitlisted patients, dialysis, kidney transplantation, depression	Cross- sectional	Sleep disorders, depressive symptoms and health related quality of life a cross sectional comparison between kidney transplant recipients and waitlisted patients on maintenance dialysis.	1067 Adults: 888 KT and 187 on waitlist, mean age 49 years	KDQoL-SF, CES-D, RLSQ, AIS, OSA	The prevalence of sleep disorders is generally less among kidney transplant recipients compared to patients on maintenance dialysis. Depressive symptoms are frequently present in patients on dialysis, and their severity and/or prevalence decreases after kidney Tx (p = 0.001). Median scores were significantly higher for the Tx vs WL groups (Physical function median 80 vs 70 p value = 0.001; General health perceptions median 50 vs 35 p value < 0.001; Energy/fatigue median 70 vs 60 p value = < 0.001; Emotional well-being median 80 vs 72 p value 0.003).
18	Mc Adams et al. (43) Baltimore	2018	Quality of Life, kidney transplantation, Frailty	prospective cohort study	Frailty and Post kidney Transplant Health-Related Quality of Life.	443 KT recipients	SF-12	Frail recipients experienced significantly greater rates of improvement in physical HRQOL (frail, 1.35 points/month; 95% confidence interval [CI], 0.65-2.05; non frail, 0.34 points/month; 95% CI, -0.17-0.85; P = 0.02) and kidney disease-specific HRQOL (frail, 3.75 points/month; 95% CI, 2.89-4.60; non frail, 2.41 points/month; 95% CI, 1.78-3.04; P = 0.01), but no difference in mental HRQOL (frail, 0.54 points/month; 95% CI, -0.17-1.25; non frail, 0.46 points/month; 95% CI, -0.06-0.98; P = 0.85) post-KT.
19	Muller et al. (44) Germany	2015	Not reported	Cross-sectional	Depression, Anxiety, Resilience and Coping Pre and Post Kidney Transplantation Initial Findings from the Psychiatric Impairments in Kidney Transplantation (PI-KT)-Study	252 adults, 101on waitlist, 151 KT, mean age 51.6	HADS-D/A, SF-12, RS FKV	The prevalence of both depressive and anxiety symptoms was not significantly different between the two groups.
20	Paterson et al. (45) Canada	2018	Adherence, renal transplant recipients	Prospective study	Medication adherence in renal transplant recipients: A latent variable model of psychosocial and neurocognitive predictors	211 underwent renal transplant at least one year prior to participation on the study	CES-D MASES-R KBIT-2 EPS CVLTII	Everyday problem solving and self-efficacy had direct positive associations with adherence. Depressive symptoms were negatively associated with self-efficacy, but not adherence. Traditionally-measured neurocognitive abilities were positively associated with self-efficacy, and negatively associated with depressive symptoms.
21	Pistorio et al. (46) Italy	2013	Not reported	Cross-sectional	The Study of Personality in Renal Transplant Patients: Possible Predictor of an Adequate Social Adaptation?	60 KT adults, Mean age 45 years	SCID-II, WHOQOL-100	The personality trait that prevailed in the female gender was borderline, while in the male gender it appeared to be predominantly obsessive compulsive personality trait.
22	Pourfarziani et al. (47) Iran	2010	Sleep, renal transplant, graft function	Cross- sectional	Assessment of sleep disturbance in renal transplant recipients and associated risk factors.	39 KT adult, Aged 18 and over	PSQI, Ifudu	67% of patients were diagnosed as “poor sleepers” (PSQI total score > or =5) and 33% were “good sleepers.” The study showed that sleep disturbance is surprisingly common in renal transplant patients.
23	Prihodova (48) Slovak Republic	2010	Neuroticism, extroversion, psychological distress, quality of life, kidney transplant, higher physical, comorbidity	Meta-analysis	Impact of personality and psychological distress on health related quality of life in kidney transplant recipients.	177 KT adults, Mean age 48 years	SF-36	Higher physical HRQoL was associated with younger age, higher education and income, a low number of comorbid diseases, lower neuroticism and distress. Higher mental HRQoL was associated with higher education and income, longer time from KT, higher extroversion, lower neuroticism and distress. In both physical and mental HRQoL, actual distress was the best predictor.
24	Raiesifar et al. (49) Iran	2014	continuous care model, quality of life, kidney transplant	randomized clinical trial	Effect of applying continuous care model on quality of life among kidney transplant patients: a randomized clinical trial.	90 KT adults, Aged 18 and over, randomly assigned to 2 groups: 41 in the experimental and 37 in the control groups.	KTQ-25	No significant difference was found between the experimental and control groups in terms of demographic variables. Although the quality of life scores increased in both groups, the mean scores of the experimental group were significantly higher than those in the control group at 1, 2, and 3 months.
25	Reilly- Spong (50) USA	2013	Not reported	Cross- sectional	Poor Sleep in Organ Transplant Recipients: Self-Reports and Actigraphy	143 KT adults, mean age 54 years	PSQI Actigraphy	41% (58 of 143) were poor sleepers (PSQI >8) and 36% used sleep medications in the past month. 15% reported having obstructive sleep apnea and 4% reported restless legs syndrome. Based on actigraphy (n = 73), 69% lacked sleep efficiency; 32% took greater than 30 minutes to fall asleep; 88% awakened during the night for more than 30 minutes; and 25% slept less than 6 hours per night. Obesity and use of psychotropics or sleep medications were independent risk factors for poor objectively-measured sleep.
26	Shabany (51) Iran	2014	medication adherence, immune-system suppressors, quality of life, renal transplant	Descriptive-correlational	Relationship between immunosuppressive medications adherence and quality of life and some patient factors in renal transplant patients in Iran.	230 KT patients, Aged 18 and over	ITAS, QoLRTxSI	There were significant correlation in: health performance (p ≤ 0.0001 & rETA = 0.23), social economic (p = 0.001 & rETA = 0.15), psychological spiritual (p = 0.011 & rETA = 0.15), also logistic test showed significant relationship between immunosuppressive medication adherence and number of transplantation (R = 1.04, p = 0.048).
27	Silva et al. (52) Brazil	2012	Quality of Life; Sleep; Renal Transplantation.	Longitudinal	The perception of sleep quality in kidney transplant patients during the first year of transplantation	76 KT adults, aged 18 and over	PSQI, SF-36; HADS-D/A, KPS	No significant differences in sleep quality between the two phases. Both the physical and mental health scores worsened from Phase 1 to Phase 2.
28	Troen et al. (53) USA	2012	Cognitive dysfunction, kidney Transplantation, depression	Cross-sectional	Cognitive dysfunction and depression in adult kidney transplant recipients: baseline findings from the FAVORIT Ancillary Cognitive Trial (FACT).	183 KT adults mean age 54 years	DSGT, TMT, CES-D	Result were: neurological or psychiatric complaints (24%); symptoms of mild to severe depression (30%); difficulty on a memory test (33%); deficit on a test of attention and mental processing speed (58%), difficulty on several tests of executive function (42%).
29	Von der Lippe (54) Norway	2014	Dialysis, Kidney transplantation, HRQOL, KDQOL-SF, Longitudinal, Clinical relevant change	Cross-sectional	From dialysis to transplantation: a 5-year longitudinal study on self-reported quality of life	110 KT adults Mean age 53,5 years	KDQOL-SF	Four of nine domains in kidney-specific HRQOL (general health, vitality, social function and role physical) improved after RTX. There were highly significant differences in HRQOL between RTX patients and the general population.
30	Wei et al. (55) Taiwan	2013	employment, kidney transplantation, long-term health related quality of life	Cross- sectional	Health related quality of life of long term kidney transplantation recipients	88 KT patients Mean age 49,1 years	MOS, SF36	The mean scores on the bodily pain (BP) subscale were the highest and, on the general health (GH) subscale, the lowest. Age, gender, serum creatinine level, and employment status were significantly related to HRQOL.
31	Weng et al. (56) USA	2013	Kidney transplantation, Epidemiology, Compliance, Adherence	Cross- sectional	Prevalence and correlates of medication non adherence among kidney transplant recipients more than 6 months post-transplant: a cross sectional study.	252 adults Mean age 54,7	ITAS, ITBS, HADS, ISEL 12, PSS-4, sTOFHLA	On the ITAS, 59.1% scored a perfect 12, 26.6% scored 10-11, and 14.3% scored 0-9. In univariate models, non-adherence (defined as ITAS score ≤9) was significantly associated with increased scores on scales for perceived stress (OR 1.12, 95% CI 1.01-1.25) and depression (OR 1.14, 95% CI 1.02-1.28), and with more self-reported barriers to adherence on the ITBS (OR 1.15, 95% CI 1.08-1.22). Stress and depression were not associated with non-adherence.
32	Zelle et al. (57) The Netherlands	2016	Physical Activity self-efficacy Renal Transplantation	Cross-sectional	Fear of Movement and Low Self-Efficacy Are Important Barriers in Physical Activity after Renal Transplantation.	487 KT adults Mean age 51 years	TSK11, LIVAS, MLTPAQ	Low physical self-efficacy and history of myocardial infarction, transient ischemic attack and cerebrovascular accident were independent determinants for fear of movement. Fear of movement was associated with lower daily PA, occupational, sports and leisure time PA. Mediation-analysis showed that a large part (73%) of the effect of fear of movement on PA was explained by low physical self-efficacy.

PSQI, Pittsburgh Sleep Quality Index; SOS, Survey of Sleep questionnaire; SCWT, Stroop Color and Word Test; DASS 21, 21-item self-report Depression; Anxiety and Stress Scale; BDI, Beck Depression Inventory; STAI, StateTrait Anxiety Inventory; MTAS, Medication Therapy Adherence Scale; GSE, General Self Efficacy Scale; SF-36, Short-Form Health Survey; SCL90R, Revised Symptom Checklist 90; TEMPS-A, Temperament Evaluation of Memphis; PSDA, Pisa and San Diego Auto-questionnaire; BAASIS, Basel Assessment of Adherence to Immunosuppressive Medication instrument; DAP, Machover Draw a Person test; EM, Electronic monitoring of non-adherence; KDQoL-SF, Kidney Disease Quality of Life; CVLTII, California Verbal Learning Test; Second Edition; D-KEFS, Delis-Kaplan Executive Function System; IADL, Instrumental Activities of Daily Living questionnaire; CIS, Checklist Individual Strength; functional impairments; SIP, Sickness Impact Profile; SLL, I/D Van Sonderen Social Support Inventory; PASE, Physical Activity Scale for the Elderly; SSS, Medical Outcomes Study Social Support Survey; CES-D, Center for Epidemiologic Studies Depression Scale; RLSQ, restless legs syndrome questionnaire; AIS, Athens Insomnia Scale; OSA, Berlin Sleep apnea questionnaire; SF-12, Short Form 12-Item Health Survey; HADS-D/A, Hospital Anxiety and Depression Scale; RS, Resilience Scale; FKV, Freiburger Fragebogenzur Krankheitsverarbeitung (Coping Self-Questionnaire); MASES-R, Medication Adherence Self-Efficacy; Scale, Revised, KBIT-2, Adherence Self-Efficacy Scale Kaufman Brief Intelligence Test; EPS, Everyday Problem Solving; SCID-II, Structured Clinical Interview Axis II Personality Disorders; WHOQOL-100, World Health Organization quality of life instrument; IFUDU, Ifudu comorbidity index; KTQ-25, kidney transplant questionnaire; ITAS, Immunosuppressive Therapy Adherence Scale; QoLRTxSI, quality of life in renal transplant patients questionnaire: satisfaction & importance; KPS, Karnofsky Performance Status scale; DSGT, Digit Symbol- Coding Test; TMT, Trail making test; CES-D, Center for Epidemiologic Studies Depression Scale; MOS, Medical Outcome Survey; ITBS, Immunosuppressive Therapy Barriers Scale; ISEL-12, Interpersonal Support and Evaluation List-12; PSS-4, Perceived Stress Scale-4; sTOFHLA, Short Test of Functional Health Literacy in Adults; TSK11, Tampa Score of Kinesiophobia-Dutch version; LIVAS, Dutch version of the Self-Efficacy for Rehabilitation Outcome Scale (SER); MLTPAQ, Minnesota Leisure Time Physical Activity Questionnaire.

**Table 2:** Low quality studies (<19).

	First author, years, country	Publication year	Key words	Study design	Title	Sample	Measure	Results
1	Ahsanuddin et al. (10) USA	2015	kidney transplantation knowledge	Cross-sectional	Candidate comprehension of key concepts in kidney transplantation	217 adult kidney transplant candidates for initial evaluation (n=100) or for reevaluation (n=117)	KAQ	Mean knowledge scores of those at evaluation (72 ± 21) and those at reevaluation (70 ± 20; p = 0.4769) were similar; therefore the entire cohort was analyzed as a single group. Compared to the high¬ scoring group, low ¬scorers (< 75%; median value) were significantly more likely to be older with lower education attainment, and have end ¬stage renal disease due to hypertension or diabetes rather than other etiologies. On multivariate analysis, independent risk factors for low¬ scores were increasing age (aOR 1.03 (95% CI 1.01¬1.06) and educational level (less than high school; aOR 4.23, 95%CI 1.82-9.80; high school or GED aOR2.85, 95% CI 1.43¬5.70 compared to some college or technical school).
2	Alvares et al. (14) Brazil	2013	Dialysis, Renal transplant, Eq5D, Quality of life	Randomized controlled trial	Factors associated with quality of life in patients in renal replacement therapy in Brazil	3036 KT adults, aged 18 and over	EQ-5D, VAS	It was observed that transplant patients have better QoL and that the most prejudicial aspects are pain/discomfort and anxiety/depression. The main factors associated with QOL are age, female gender, variables associated with the clinical condition of the patient such as the need for hospitalization and the presence of comorbidities, social class and variables associated with the health service use.
3	Ay et al. (4) Turkey	2015	Control Groups, Kidney Transplantation, Living Donors, Quality of Life	Longitudinal	Evaluation of Quality of Life Early and Late After Kidney Transplantation.	141 adults, 47 donors (mean age 41,23), 47 KT recipients (mean age 32,38), 47 healthy controls (mean age 38,2)	SF 36	Physical functioning (PF) was higher at the 9th postoperative month compared to the 3rd month in the recipient group (p = 0.028). Donors had higher PF (p= 0.007) and functioning physical role (PR; p = 0.01) compared to recipients. Recipients had lower PF (p = 0.016), PR (p = 0.004), and functional-emotional role (ER; p = 0.03) at the 3rd month and had lower PR (p = 0.002) at the 9th month postoperatively comparing to the control group. Donors had lower PF (p = 0.007) and PR (p = 0.01) at the 3rd month and had lower PR (p = 0.035) at the 9th month postoperatively comparing to the control group. Donors and recipients had similar QOL at the 9th month.
4	Bello et al (7) Nigeria.	2016	Knowledge belief chronic kidney disease	Cross- sectional	Knowledge, attitudes and beliefs of first-degree relatives of patients with chronic kidney disease toward kidney donation in Nigeria.	161 adults, mean age 24,8	self-administered questionnaire to study the relatives of patients with CKD	About 85.1% of the respondents were aware that kidney transplantation was a treatment option for end stage renal failure, while 70% of them believed that kidney transplantation resulted in an improvement in the quality of life of these patients. However, 25.5% of the respondents believed that kidney donors were at risk of developing kidney failure in the future. Overall, 77.6% of the respondents were willing to donate a kidney, especially if the affected individual was their offspring. The majority of the respondents were willing to donate a kidney to a relative with CKD.
5	Burkhalter (58) Switzerland	2016	Adults; chronobiology; kidney transplantation; melatonin profile; rental transplant recipients; salivary melatonin; sleep-wake disturbances	Randomised, multicentre wait-list controlled trial	Melatonin rhythms in renal transplant recipients with sleep-wake disturbances.	KT recipients [n = 29 (aged 54.83 ± 13.73)] were retrospectively grouped into two groups: 11 whose dim light melatonin onset (DLMO) could be calculated and 18 whose DLMO could not be calculated	PSQI; ESS; MEQ; DASS	RTx recipients having a measurable dim light melatonin onset (DLMO) had a number of differences from those without DLMO: they were younger [46.4 ± 14.9 compared to 60.0 ± 10.3 (p = .007)], had higher hemoglobin values [135.36 ± 12.01 versus 122.82 ± 11.56 (p = .01)], less anxiety [4 (0;8) versus 12 (6.5;14) (p = .021)] and a better overall sense of coherence [SOC Score: 71.09 ± 12.78 versus 56.28 ± 15.48 (p = 0.013)]. These results suggest that RTx recipients whose DLMO could be calculated have less health impairments, underlying the relevance of a stable circadian system.
6	Costa-Requena et al. (59) Spanish	2017	Depression; Health related quality of life; Longitudinal; Renal transplantation; Specific-symptoms of kidney disease	longitudinal study	Health related quality of life in renal transplantation: 2 years of longitudinal follow-up	Renal Transplant recipients	SF 36, HADS	At 6-months after transplantation, patients had similar HRQoL scores compared to the general population. The improvement on effects of kidney disease domain could be considered as large (η^2^ = 0.29), and medium on burden of kidney disease domain (η^2^ = 0.12), work status domain (η^2^ = 0.12), and sexual function domain (η^2^ = 0.13). Psychological distress, depressive symptoms, hemoglobin, and serum creatinine had significant influence on patient's perceived HRQoL over 2 years after transplantation.
7	Czyzewski et al. Poland (60)	2018	Quality of Life, Depression, Anxiety, Stress Kidney Transplantation.	Cross- sectional	Evaluation of Quality of Life and Severity of Depression, Anxiety, and Stress in Patients After Kidney Transplantation.	118 post-KT patients	SF-36, KDQOL, DASS	Correlation analysis showed a statistically significant influence of age on general health (R = 0.191, P = .039), physical functioning (R = -0.295, P = .001), and general physical health (R = -0.275, P =.003) assessment. The mean severity of depression, anxiety, and stress among subjects changed over time since KTx. For the post-KTx periods studied (ie, < 1 year, 1-10 years, and >10 years), the following changes were observed: for depression, 14.0 vs 11.2 vs 13.1, respectively; for anxiety, 15.6 vs 9.8 vs 14.0, respectively; and for stress, 22.0 vs 13.5 vs 16.8, respectively.
8	De Pasquale et al. (24) Italy	2014	Psychiatric consultation; Psychological care; Kidney transplantation; Therapeutic compliance; Social and family support	Review	Psychopathological aspects of kidney transplantation: Efficacy of a multidisciplinary team	Not Reported	Not reported	Integrated and multidisciplinary care should include uniform criteria and procedures for standard assessments, for patient autonomy, adherence to therapy, new coping strategies and the adoption of more appropriate lifestyles.
9	De Pasquale et al. (61) Italy	2011	Quality of life, kidney transplantation, post-transplant, social activities, vitality perceived health	Cross-sectional	Quality of life in kidney transplantation from marginal donors.	70 kidney Transplantations adults, aged 55 and over	SF-36	Donor age did not negatively influence health status perceived by the subjects. The vitality and mental health seemed to increase with greater donor ages, but the status of perceived health, vitality, social activities, and mental health were negatively influenced by the age of the transplant.
10	Famà et al. (62) Italy	2013	Health-related quality of life, Kidney transplant, Epidemiological study	Longitudinal	Social and interpersonal relationship modifications after renal transplant. A statistic and epidemiologic evaluation	75 adults mean age 52,6 years	SF-36	The benefits obtained on quality of life after transplantation is the prerogative of a small percentage of patients and is related to medium and high socioeconomic conditions. The possibility of avoiding the hemodialysis represents the primary benefit for the totality of patients
11	Hucker et al. (63) UK	2017	Adherence kidney transplant	Systematic review	Non-adherence to immunosuppressants following renal transplantation: a protocol for a systematic review	Post-transplant recipients with graft survival and graft failure	PRISMA guidelines; Downs and Black checklist	This review aims to summarize what is known about non-adherence, with a view to providing comprehensive evidence to inform on strategies aimed at advancing adherent behavior.
12	Husain et al. (64) USA	2018	Scientific Registry for Transplant Recipients (SRTR); clinical research/practice; health services and outcomes research; kidney transplantation/nephrology; organ procurement organization; patient characteristics; quality of care/care delivery; social sciences	observational	Patients prioritize waitlist over post-transplant outcomes when evaluating kidney transplant centers	409 respondents, of whom 68% were KT recipients and 32% had chronic kidney disease or were on dialysis. Participants had mean age 56 ± 12 years.	anonymous electronic survey of patients that assessed the relative importance of patient experience, practical considerations, transplant center reputation, center experience, and waitlist when selecting a transplant center.	Participants most often prioritized waitlist when evaluating transplant centers (transplanted 26%, chronic kidney disease 40%), and waitlist was almost twice as likely as outcomes to be ranked most important (30% vs 17%). Education level and transplant status were significantly associated with factors used for center prioritization. Waitlisted respondents most commonly (48%) relied on physicians for information when selecting a center, while a minority cited transplant-specific organizations. In order to improve shared decision-making, materials outlining center- specific waitlist features should be prioritized. Novel patient-oriented metrics for measuring transplant center quality that align with patient priorities must be explored.
13	Kung et al. (65) Taiwan	2017	Not reported	cross-sectional	Renal Transplant Recipients: The Factors Related to Immunosuppressive Medication Adherence Based on the Health Belief Model	122 KT patients	A self-developed questionnaire regarding the personal characteristics and health-related beliefs of patients on adherence to treatment with immunosuppressive medication.	Participants who had received dialysis treatment or had experienced rejection perceived susceptibility to rejection more strongly than those who had not. Participants who had undergone transplantation, had experienced more drug-related symptoms, or had contracted severe to extremely severe infections in the past showed lower rates of adherence to treatment with immunosuppressive medication. Adherence to medication regimens correlated negatively with length of time since transplantation. Length of time since transplantation, drug-related symptoms, perceived susceptibility to rejection, and perceived benefits of treatment were identified as major predictors of adherence to immunosuppressive medication regimens.
14	Liaveri et al. (66) Greece	2017	Blood pressure; Hemodialysis; PTSD; Renal transplantation; Sleep quality	cross-sectional	Quality of sleep in renal transplant recipients and patients on hemodialysis	KT recipients (n=152), patients on hemodialysis (n=67) and healthy participants (n=49)	AIS	The highest mean AIS score was observed in the transplant patients (KTx: 4.6 ± 13.3 vs. HD: 3.8 ± 8.1 vs. NOR: 2.4 ± 10.2); both KTx and HD patients had a lower quality of sleep compared to participants with normal renal function.
15	Neri et al. (67) Italy	2012	Kidney transplantation, Chronic kidney disease, Quality-adjusted life years, Kidney function, Self-reported outcomes	cross-sectional	Characterizing the relationship between health utility and renal function after kidney transplantation in UK and US: a cross-sectional study	1251 KT patients, of them 157 were on continuous ambulatory peritoneal dialysis, 268 were on hemodialysis,115 were on CKD pre dialysis stage, and 711 received a transplant	EQ-5 D	CKD severity was negatively associated with EQ-5D index in both samples (UK: ρ = −0.20, p = 0.02; US: ρ = −0.21, p = 0.02). The mean adjusted disutility associated to CKD stage 5 compared to CKD stage 1–2 was Δ = −0.38 in the UK sample, Δ = −0.11 in the US sample and Δ = −0.22 in the whole sample. The adjusted median disutility associated to CKD stage 5 compared to CKD stage 1–2 for the whole sample was 0.18 (p < 0.01, quantile regression). Center effect was not statistically significant.
16	Panagopoulou et al. (68) Greece	2009	renal transplantation, quality of life anxiety, depression, Hemodialysis	Cross- sectional	Psychosocial issues and quality of life in patients on renal replacement therapy	124 adults, 40 patients undergoing in center hemodialysis patients, 36 continuous ambulatory peritoneal dialysis, and 48 renal transplant recipients, mean age 51,66	Questionnaire examining demographics, functional status, employment status, and impact of therapy on psychosocial issues such as anxiety and depression.	The RTx patients disclosed a better functional and employment status than the CAPD and the HD patients. They were also more compliant and satisfied with their therapy and their relationship with the medical and nursing personnel. The CAPD patients were also more satisfied, more compliant, better motivated, and less anxious and depressed compared with the HD patients who scored low in every aspect studied.
17	Pistorio et al Italy (20)	2017	Not reported	Cross-sectional	Alexithymia in Kidney Transplantation Patients	32 kidney transplantation patients from a deceased donor, mean age 53,06	TAS, SCL-90 R SF-36	The study showed a high percentage of the presence of alexithymia in the examined transplant recipients where the perception of their quality of life is low and where there is a greater presence of psychosomatic symptoms.
18	Pistorio et al. (21) Italy	2015	Kidney transplantation, thematic apperception test, quality of life, emotional affective aspects	Cross-sectional	A Study on Emotional-Affective Aspects and the Perception of General Health in Kidney Transplant Subjects.	30 KT adults, Mean age 44,5	TAT, SF-36	The variable of TAT “communication difficulties” was significantly correlated with the dimensions of the SF36 “emotional role functioning,” “social activities,” and “mental health.” The dimension of SF36 “general health” did not interfere with the variable of TAT “constricted effect.”
19	Ronai et al. (69) Hungary	2017	Beta activity Insomnia Kidney transplant recipients Polysomnography Sleep architecture Slow wave sleep	Cross-sectional	Association of symptoms of insomnia and sleep parameters among kidney transplant recipients	100 KT adults	AIS, polysomnography with power spectral analysis.	In univariable analysis AIS score was not associated with sleep macrostructure parameters (sleep latency, total sleep time, slow wave sleep, wake after sleep onset), nor with NREM and REM beta or delta activity in sleep microstructure. In multivariable analysis after controlling for covariables AIS score was independently associated with the proportion of slow wave sleep (β = 0.263; CI: 0.026-0.500) and REM beta activity (β = 0.323; CI = 0.041-0.606) (p < 0.05 for both associations).
20	Sieverdes et al. (70) Carolina	2015	kidney transplant dialysis physical activity	Randomized controlled trial	Attitudes and perceptions of patients on the kidney transplant waiting list toward mobile health delivered physical activity programs.	22 dialysis patients, mean age 46 years, mean duration on transplant waiting lists was 6.7 years	COREQ-32	High interest (95%) for using to promote physical activity was found. Spirituality provided strength to engage in physical activity. Patients preferred their home and neighborhood environments to intra-dialytic settings for engaging in physical activity.
21	Srifuengfung et al. (71) Thailand	2017	Not reported	cross-sectional, observational	Depression in Kidney Transplant Recipients: Prevalence, Risk Factors, and Association With Functional Disabilities	217 consecutive kidney transplant (KT) recipients	CCI PHQ-9 WHODAS	Twenty-eight (12.9%) patients had depression (PHQ-9 score, ≥10). A binary logistic regression analysis found that the CCI score was significantly higher in KT recipients with depression (β = 0.54, p < 0.01). After the adjustment of education and glomerular filter rates, an ordinal logistic regression analysis revealed that the PHQ-9 scores were positively correlated with the WHODAS scores (β = 0.39, p < 0.01). In KT recipients, physical comorbidity is associated with depression, and depression is correlated with functional disability.
22	Szeifert et al. (72) Hungary	2010	Depression, kidney transplant, waiting list, comorbidity	Cross- sectional	Symptoms of depression in kidney transplant recipients: a cross-sectional study.	1030 adults, 854 kidney transplant and 176 on waitlist	CESD	The prevalence of depression was 33% versus 22% in waitlisted versus transplant patients, respectively (P = 0.002). In multivariate regression, number of comorbid conditions, estimated glomerular filtration rate, perceived financial situation, and marital status were significant and independent predictors of depression in the transplant recipient group. The prevalence of depression is lower in transplant recipients than in waitlisted patients (OR, 2.01; 95% CI, 1.253.23; P = 0.004).
23	Tamura et al. (73) Japan	2018	Mood Status, Quality of Life, Kidney transplant Recipients	Descriptive Correlational Study	Mood Status and Quality of Life in Kidney Recipients After Transplantation.	Sixty-eight post-kidney transplant patients	SF-36 Version 2	The QOL of the transplantation group was significantly higher for all 8 subscales of SF-36 compared to the hemodialysis group. Among the factors, greater age and higher Confusion levels were related to lower physical QOL. In addition, higher Vigor and lower Fatigue levels were related to higher mental QOL, while the condition of having an occupation was related to higher role/social QOL.
24	Tavallaii et al. (74) Iran	2009	Quality of Life, Anxiety, Depression, Kidney Transplant	Cross-sectional study	Socioeconomic Links to Health-Related Quality of Life, Anxiety, and Depression in Kidney Transplant Recipients	242 kidney transplant recipients	SF-36	The study showed that kidney allograft recipients with a low income had a poorer health related quality of life and a greater load of anxiety according to their perception of their status, compared to those with higher incomes. The depression symptom scores were not significantly different between the income groups. Logistic regression analysis showed that the impact of income on the total HRQL and anxiety symptoms scores remained significant after controlling the effect of age, sex, and time interval from transplantation.
25	Van Lint et al. (75) Netherlands.	2017	Self-monitoring, renal transplant recipients	Randomized controlled trial	Self-Monitoring Kidney Function Post Transplantation: Reliability of Patient-Reported Data.	54 renal transplant patients	StatSensor Xpress-i Creatinine Meter for self-monitoring Creatinine and Microlife WatchBP Home for self-monitoring blood pressure	The results of the study showed the non-safety of entrusting electronic self-monitoring measures to patients. This should be well considered when designing self-monitoring systems, for example by ensuring that self-measured data is automatically transferred to an Web-based Self-Management Support System (SMSS), which provided automatic feedback on the registered values (eg, seek contact with hospital).
26	Wang et al. (76) The Netherlands	2017	Renal transplant patient; Self-management; Survey; Technology acceptance; e-health	cross-sectional, observational	Renal transplant patient acceptance of a self-management support system.	fifty KT patients	SMSS	Self-management support systems (SMSS) have been proposed for renal transplant patients to increase their autonomy and reduce the number of hospital visits. Results showed that some patients would like to continue using the SMSS after 1 year, others no longer felt the need to use the SMSS. In the first few months post-transplantation, only a limited number of outpatient visits was replaced by a telephonic consult.
27	Weng et al. (77) Taiwan	2017	health care; immunosuppressant; kidney transplantation; medication adherence; medication belief; non adherence; nursing; partnership; self-efficacy; symptom distress	cross-sectional and correlation design	Factors that determine self-reported immunosuppressant adherence in kidney transplant recipients: a correlational study.	145 KT recipients	Structured questionnaires to assess adherence to immunosuppressant treatment	The results of the multivariate linear regression analysis showed that gender (male), low income with a high school or college education, years after transplantation and concerns about medication taking were negatively associated with adherence. Medication self-efficacy was positively associated with adherence. Therapy related factors, partnerships with healthcare professionals and having private healthcare insurance did not significantly relate to immunosuppressant therapy adherence.
28	Xie et al. (78) China	2018	Sleep quality, renal transplant	cross-sectional study	Sleep Quality and Related Psychosocial Variables Among Renal Transplant Patients	485 renal transplant patients	PSQI, TICS, FS	Significant differences in the PSQI scores were observed in renal transplant patients of different sex, age, residence, career, length of post-renal transplant period, comorbidity, kidney function, and depressive symptoms (P < .05). The global PSQI score was 5.86 (SD, 3.20), significantly lower than the norm; The PSQI scores were positively correlated with depression (Self-Rating Depression Scale) (P < .01) but negatively correlated with psychological well-being (Flourishing Scale) (P < .01).
29	Zhao et al. (79) China	2018	Renal transplant recipients, quality of life, adherence Social Support	Descriptive Correlational Study	Quality of Life, Adherence Behavior, and Social Support Among Renal Transplant Recipients in China: A Descriptive Correlational Study	253 KT recipients	Structured questionnaires to assess quality of life, adherence behavior, and social Support	Time since transplantation (P = .041) and education (P = .013) were factors affecting QoL scores. Occupation (P = .0000087), marital status (P = .013), payment method (P = .028) and monthly income (P = .007) affected the total social support score; there were also significant relationships between physical health, psychological health, adherence behavior (r = .145, P = .022; r = .153, P = .016), and social support (r = .211, P = .001; r = .301, P = .000).
30	Zhu et al. (80) China	2017	Immunosuppression; Kidney Transplantation	meta-analysis and systematic review	Efficacy of interventions for adherence to the immunosuppressive therapy in kidney transplant recipients: a meta-analysis and systematic review.	Eight studies were included with a total for 546 patients	Cochrane Collaboration's tool for assessing risk to assess the included studies; the statistical software Comprehensive Meta-Analysis, V.2.0 for statistical analyses	Sensitivity analysis indicated that findings for adherence rate were robust. Among participants receiving intervention, the adherence rate was significantly higher than the control group (pooled OR=2.366, 95% CI 1.222 to 4.578, p = 0.011). Intervention programs designed to increase immunosuppressive adherence in patients with kidney transplant improve treatment adherence.

KAQ, Knowledge Assessment Questionnaire; VAS, Visual Analog Scale; EQ-5D, Euro Qol 5 dimension index; SF36, Health Survey 36 items; PSQI, Pittsburgh Sleep Quality Index; EES, Epworth sleepiness scale; MEQ, Morningness; Eveningness Questionnaire; DASS, Depression; Anxiety and Stress Scale; HADS, Hospital Anxiety and Depression Scale; KDQoL, Kidney Disease Quality of Life; PRISMA, Preferred Reporting Items for Systematic Reviews and Meta-analyses; SAHS, Diagnosis of sleep apnea hypopnea syndrome; AIS, Athens Insomnia Scale; TAS- 20, Toronto Alexithymia Scale; SCL90R, Revised Symptom Checklist 90; TAT, Thematic Apperception Test; COREQ-32, Consolidated Criteria for Reporting Qualitative Studies 32-Item Checklist; CES-D, Center for Epidemiologic Studies Depression Scale; SMSS, Web-based Self-Management Support System; SMSS, Web-based Self-Management Support System; TICS, Three-dimensional Inventory of Character Strengths; FS, Flourishing Scale.

**Table 3:** Results of quality assessment using Downs and Black checklist.

First author (ref.)	Study reporting	External validity	Internal validity, bias	Internal validity confounding	Power	Quality score*
Ahsanuddinl (10)	1,2,3,4,5,6,7,10	11,12,13	18,19,20	21,22,25	27	18
Alvares (14)	1,2,3,6,7,10	11,12,13	17,18,20	21,22,23	27	16
Ay (4)	1,2,3,6,8,10	11,12,13	17,18,20	21,22,23	27	16
Bello (7)	1,2,3,4,5,6,7,10	11,12,13	18,19,20	21,22,25	27	18
Burkalter (58)	1,2,3,4,5,6,7,10	11,12,13	17,18,19,20	22,23,25	--	18
Burkhalter (28)	1,2,3,4,5*,6,10	11,12,13	17,18,19,20	21,22,23,25	27	20
Burkhalter (29)	1,2,3,4,5,6,7,9, 10	11,12	16,17,18,19,20	21,22,23,24,25,26	27	23
Corruble (30)	1,2,3,4,5*,6,8,9,10	11,12,13	17,18,19,20,	21,22, 26	27	21
Costa-Requena (59)	1,2,3,6,7,8,10	11,12	18,20	21,22,23,26	27	16
Cukor (31)	1, 2, 3, 4, 6, 7, 10	11, 12, 13,	17, 18, 19, 20	21, 22, 25, 26	27	19
Czyzewski (60)	1,2,3,4,5,6,7,8,10	11,12,13	17,18,19,20	21,22,25	--	18
De Pasquale (16)	1,2,3,5,6,7,8,9,10	11,12,13	17,18,20	21,22,26	27	19
De Pasquale (24)	1,2,3,6,7,8,10	11,12,13	17,18,20	21,22,23,26	27	18
De Pasquale (32)	1, 2 ,3,5,6 ,7,8,9,10	11, 12, 13,	17, 18, 20	21, 22, 26,	27	19
De Pasquale (61)	1,2,3,6,7,8,10	11,12,13	17,18,20	21,22,23,26	27	18
De Pasquale (9)	1, 2, 3, 4, 6, 8, 9, 10	11, 12, 13	17, 18, 19, 20	21, 22, 26	27	19
Denhaerynck (33)	1, 2, 3, 4, 6, 7,9,10	11, 12, 13	15, 17, 18, 19, 20	21, 22, 23,26	27	21
Famà (62)	1,2,3,6,7,10	11,12,13	17,18,19,20	21,22,23,26	27	18
Fructuoso (34)	1, 2, 3, 4, 5*, 6, 10	11, 12, 13	17, 18, 19, 20	21, 22, 23, 25	27	20
Gelb (35)	1,2,3,4,5*,6,7,9,10	11,12,13	17,18,19,20	21,22,23,	27	21
Gentile (36)	1, 2, 3, 6, 7, 8, 9, 10	11, 12, 13	17, 18, 19, 20	21, 22, 23, 26	27	20
Gheith (37)	1,2,3,4,5*,6,8,9,10	11,12,13	17,18,19,20,	21,22, 26	27	21
Goedendorp (38)	1,2,3,4,6,7,10	11,12,13	15,17,18,19,20	21,22,23	27	19
Gordon (39)	1, 2, 3, 4,5,6, 7, 9, 10	11, 12, 13	17, 18, 19,20	21, 22, 26	27	20
Gross (40)	1, 2,3,4,5*, 6, 7, 8, 9, 10	11, 12, 13	16, 17, 18, 19, 20	21, 22, 23, 24, 25, 26	27	26
Hucker (63)	1,2,3,4,5,6,7,10	11,12,13	17,18,19,20	22,23,25	--	18
Husain (64)	1,2,3,4,6,7,10	11,12,13	18,19,20	21,22,	27	16
Kofman (41)	1,2,3,4,5,6,7,8,9,10	11,12,13	17,18,19,20	21,22,23,25,26	27	24
Kovacs (42)	1,2,3,6,7,9,19	11,12,13	15,17,18,19,20	21,22,23,25,26	27	21
Kung (65)	1,2,3,4,6,10	11,12,13	18,19,20	21,22,25,26	27	17
Liaveri (66)	1,2,4,6,7,10	11,12,13	18,19,20	22,25,26	27	16
Mc Adams (43)	1,2,3,4,5,6,7,10	11,12,13	16,17,18,19,20	21,22,23,25,26	27	22
Muller (44)	1,2,3,5,6,7,9,10	11,12,13	17,18,19,20	21,22,23,24,25,26	27	22
Neri (67)	1,2,3,4,6,9,10	11,12	18,19,20	21,22,26	27	16
Panagopoulou (68)	1,2,3,6,7,10	11,12,13	16,17,18,20	21,22,25	27	17
Paterson (45)	1,2,3,4,5,6,7,8,9,10	11,12,13	16,17,18,19,20	21,22,23,25,26	27	24
Pistorio (46)	1, 2, 3,5, 6,7, 8, 10	11, 12, 13	17, 18, 19, 20	21, 22, 25	27	19
Pistorio (20)	1,2,3,5,6,7,8,9	11,12,13	18,19,20	22,25	27	17
Pistorio (21)	1, 2, 3,5, 8, 10	11, 12, 13	17, 18, 19, 20	21, 22, 25	27	17
Pourfarziani (47)	1,2,3,5*,6,7,10	11,12,13	17,18,19,20	21,22,26	27	19
Prihodova (48)	1, 2, 3, 5*, 6, 7, 10	11, 12, 13	17, 18, 19, 20	21, 22, 26	27	19
Raiesifar (49)	1, 2, 3, 4, 6, 7, 10	11, 12, 13	15, 17, 18, 19, 20	21, 22, 23	27	19
Reilly-Spong (50)	1,2,3,4,5*6,7,8,9,10	11,12,13	16,17,18,19,20	21,22,23,25,26	27	25
Ronai (69)	1,3,5,6,8,9,10	11,12	16,18,19	22,23,25	27	16
Shabany (51)	1, 2, 3, 4, 6, 7, 9, 10	11, 12, 13	18, 19, 20	21, 22, 25, 26	27	19
Sieverdes (70)	1,2,3,4,6,7,10	11,12,13	18,19,20	21,22,25	27	18
Silva (52)	1,2,3,6,7,9,10	11,12,13	17,18,19,20	21,22,23,24,25,26	27	21
Srifuengfung (71)	1,2,3,6,7,8,10	11,12	16,18,20	22,23,26	27	16
Szeifert (72)	1,2,3,6,7,10	11,12,13	17,18,20	21,22,23	27	16
Tamura (73)	1,3,6,8,10	11,12,13	18,19,20	21,22,25	27	15
Tavallaii (74)	1,2,3,6,9,10	11,12,13	17,18,19,20	21,22,23	27	17
Troen (53)	1,2,3,4, 5^*^, 6, 7, 9, 10	11, 12, 13	14, 15, 16, 17, 18, 19, 20	21, 22, 23, 24, 25, 26	27	27
Van Lint (75)	1,2,3,4,6,7,10	11,12,13	17,18,19,20	21,22,23,26	--	18
Von Der Lippe (54)	1, 2, 3,5,6, 7, 9, 10	11, 12, 13	17, 18, 19, 20	21, 22, 26	27	19
Wang (76)	1,2,3,4,7,9,10	11,12,13	18,20	23,25,26	27	16
Wei (55)	1, 2, 3, 5,6, 7,8, 9, 10	11, 12, 13	17, 18, 20	21, 22, 26	27	19
Weng (56)	1,2,3,4,5*,6,8,9,10	11,12,13	15,17,18,19,20	21,22,26	27	22
Weng (77)	1,2,3,4,6,7,8,9,10	11,12,13	18,19,20	25,26	27	18
Xie (78)	1,2,3,4,5,6,7,10	11,12,13	18,19,20	21,22,25	27	18
Zelle (57)	1,2,3,4,5*,6,10	11,12,13	17,18,19,20	21,22,23,25	27	20
Zhao (79)	1,2,3,4,5,6,7,10	11,12,13	18,19,20	21,22,25	27	18
Zhu (80)	1,3,6,7,8,9,10	12,13	18,19,20	23,25,26	27	16

*Item 5 = 2.

#### Quality Score and Quality Differentiation

Performing a meta-analysis seemed inappropriate due to the heterogeneity of the included studies, the authors decided pragmatically to categorize the outcome measures in outcome domains on the basis of consensus among study authors (CDP, MLP, MV, AG, PV) and they sorted two domains according to the criteria of the SIPAT (Stanford Integrated Psychosocial Assessment for Transplantation): “patients’ readiness level and illness management;” “psychological stability and psychopathology” (SIPAT domains 1 and 3) (27). These two domains represent the most important aspects of recent literature on deceased donor kidney transplantation, psychosocial factors and patient’s therapeutic adherence.

The quality of the studies was assessed independently by the CDP, MLP, LI reviewers using the “Downs and Black checklist for non-randomized studies” (81). The Downs and Black checklist is composed of 27 items distributed over five sub-scales: “reporting” (10 items), which verifies that the data in the document comes from an impartial evaluation of the results of the study; “external validity” (3 items), which measures the possibilities of generalization of the study to the reference population; “bias” (7 items), which measures the possibility of “bias” in the results of the study; “confounding” (6 items), which dealt with the “bias” in selecting the study participants; “power” (1 item), which allows to evaluate possible study errors due to chance. Responses are assigned 0 or 1, with the exception of item 5, which can get a score from 0 to 2. The maximum total score was therefore 28 (81). Since the checklist is not a classification to distinguish between low and high quality studies, consequently we defined a study with a score ≥ 19 a *priori* as being considered of high quality, reflecting two-thirds of the maximum score, such as reported in the literature (82).

## Results

### Number of Studies Found in the Selection of Studies

The PUBMED search generated 1,332 articles and 838 additional articles were found through the Cochrane, Psychoinfo, Scopus, Embase databases. The combined search strategy collected a total of 1,109 articles after removing duplicates. 137 full-text articles were evaluated for suitability with respect to inclusion and exclusion criteria after the exclusion of 972 registrations after the revision of titles and abstracts. Of these, 75 were removed after reviewing the full-text articles, as they did not contain primary quantitative data, contained duplicate data or otherwise fulfilled the exclusion criteria (**Figure 1**). Therefore, 62 articles were admitted in the systematic review. In **Table 1** we reported the high quality studies (score ≥ 19), presented in detail in the text. **Table 2** shows the low quality studies (score ≤ 19).

### Quality Score Results

The results of the quality assessment using Downs and Black checklists are shown in **Table 3**. Thirty-two articles were classified as high quality with an average score of 20.91; fifteen concerned patients’ readiness level and illness management with an average total score of 19.67; seventeen studies concerned psychological stability and psychopathology with an average total score of 22.

#### Patient’s Readiness Level and Disease Management

A total of 30 studies investigated patient’s readiness level and illness management, 15 of them were high-quality studies and will be described.

#### Personality Factors and Therapeutic Adherence

Adherence to post transplant immunosuppressive therapy is of fundamental importance for the long-term survival of the graft. Personality factors can diversify the emotional response to transplantation and the clinical course of post-transplant patients. A wider perception of the personality of these patients is essential for favorable outcomes for kidney transplantation.

The study of Pistorio et al. (46) shows that personality traits, such as borderline (BP) and obsessive-compulsive (OCP), are predictors of low social adaptation in transplanted patients. The authors explored personality traits in 60 deceased donor kidney transplant recipients, with the “Structured Clinical Interview Axis II Personality Disorders” (SCID-II) for “Diagnostic and Statistical Manual of Mental Disorders fourth edition” (DSM-IV R). The OC personality trait and quality of life correlate negatively (Pearson r correlation) (r = -0.401; p < 0.05). The obsessive personality trait was more present in the male subjects, while the borderline personality trait was found more in the female subjects. These subjects showed a low level of general health (r = -0.188; p < 0.05) and difficulties in interpersonal relationships (r = -0.147; p < 0.05). The study suggests that personality traits are a good indicator of quality of life. In this study, early identification, with adequate assessment, of subjects transplanted with behavioral problems is required to provide them with adequate psychological-psychiatric support during follow-up.

Prihodova et al. (48) studied the impact of some personality factors (neuroticism, extroversion and psychological stress) on the “health-related quality of life” (HRQOL) of 177 patients with renal transplantation. “Short Form Health Survey” (SF-36) was used to study quality of life. Personality factors were studied using the Eysenck Personality Questionnaire Revised-Abbreviated (EPQR-A) and to identify psychological stress, the General Health Questionnaire-12 (GHQ-12) was used. Data analysis showed that high scores in the physical component of quality of life are associated with young age (p value ≤ 0.001), higher education level (p value ≤ 0.05) and low scores on the “neuroticism” factor (p value ≤ 0.005) and psychophysical stress (p value ≤ 0.001). High scores on the SF-36 mental health index were associated with a greater presence of extraversion (p value ≤ 0.05) and a low presence of neuroticism (p value ≤ 0.001) and psychological stress (p value ≤ 0.001). In summary, the study shows, with reference to the literature data, that high psychological stress can cause low quality long-term life in transplanted subjects and contributes considerably to the presence of somatic symptoms in these patients.

The study of Cukor et al. (31) compared patients with end-stage renal disease (ESRD) including both kidney transplant recipients and subjects undergoing hemodialysis (HD) treatment on psychological-emotional evaluations and adherence to therapeutic treatments. Ninety-four patients undergoing functional renal transplantation and 65 hemodialysis patients completed the evaluation (total participants 159). The questionnaires used were: Beck Depression Inventory-II (BDI) to measure possible depression; Immunosuppressive therapy adherence scale-medication (ITAS-M) to assess adherence to immunosuppressive therapy.

The results showed that a greater presence of depressive symptoms corresponded to a lower therapeutic adherence in the sample of hemodialysis subjects (r = -0.47, p value = 0.001), in the sample of transplanted subjects (r = -0.38, p value = 0.001) and in the whole sample (r = -0.47, p value = 0.001).

In a prospective study, Denhaerynck et al. (33) assessed and comprehensively tested the possible risk factors for nonadherence in 249 kidney transplant patients using cutting-edge measures. This study included patients who had undergone kidney transplantation for at least one year, over the age of 18 years. Non-adherence to immunosuppressive therapy was assessed using an electronic measuring instrument: MEMS^®^-V Track-Cap (Aardex Group, Switzerland).

Some significant variables were examined, potentially related to a good therapeutic adherence: self-efficacy with the use of drugs, using the Long-term Medication Behaviour Self-Efficacy Scale; representations of the disease, according to the Siegal scale of 10 elements; The coping was evaluated by the Utrecht Coping List (UCL); The authors also measured depressive symptoms using the Beck Depression Inventory. The results of the study showed that the most significant risk factors are: young age [odds ratio (OR) = 0.96; q-value (q) = 0.02], male gender (OR = 0.37; q value = 0.02), low self-efficacy (OR = 0.24; q value = 0.02), high self-reported non-adherence (OR = 4.34; q value < 0.001), hectic lifestyle (OR = 2.3; q value = 0.03). To conclude, the authors underline how important it is to analyze the variables useful for detecting non-compliance or to improve adherence.

De Pasquale et al. (16) evaluated the possible psychopathological variables responsible for non-adherence behaviors in 74 kidney transplant patients. For the psychological-psychiatric evaluation the following tests were used: Symptom checklist 90—revised (SCL-90 R) to evaluate the psychological symptomatology, Temperament Evaluation of Memphis, Pisa and San Diego Questionnaire (TEMPS-A) for the study of the personality and temperament, the Short Form Health Survey (SF-36) to assess mental health score (MIS), Basel Assessment of Adherence to Immunosuppressive Medication instrument (BAASIS) for assessing adherence to the immunosuppressive drug. Analysis of the data showed that the subjects most educated and transplanted the longest had good level of mental health “(Education, E/MIS r = 0.61; Years from transplantation, YT/MIS r = 0.48).” The female gender was less adherent to therapy, while the perceived state of mental health did not affect adherence “(MIS/BAASIS total score, BT r = -0.01).” The cyclothymic (CT), irritable (IT), and depressive (DT) temperament was negatively correlated with therapeutic adherence “(BT/CT r = 0.39; BT/IT r = 0.44; BT/DT r = 0.21).” Significant associations (p value < 0.05) emerged among some predictors (gender, post-transplant years, anxiety and cyclothymic temperament) and the outcome variable (BT). Through this study, the authors proposed cognitive pathways and psychotherapy for a better adaptation of transplanted subjects and to guarantee a good post-transplant quality of life. In this context, the study of post-transplant quality of life becomes essential.

#### Self-Perceived Health and Post-Transplant Lifestyles

In kidney transplanted subjects it is important to deepen the perception of one's health. The subject in the immediate post transplant must be helped to plan self-efficacy and resilience skills, necessary to achieve a correct lifestyle for the maintenance of the transplanted organ.

Fructuoso et al. (34) examined the concept of quality of life, subjectively perceived, in 4 groups of patients: thirty of the 821 patients with chronic kidney disease (CKD) in stage 1–4, 30 of 117 kidney transplanted patients (KT), 37/43 in hemodialysis patients (HD) and 14/17 in peritoneal dialysis (PD). Two tests were administered to the subjects: short Form-36 (SF-36) and “kidney disease and quality of life—short form” (KDQOL-SF). The four groups achieved better results on the “social functioning” scale “(77.68 ± 18.46 in PD; 74.17 ± 29.53 in KT; 66.81 ± 31.39 in CKD 1 -4; 62.16 ± 32.84 in HD; p value = 0.192).” Non-significant scores were found in the “General Health” scale of the physical health component “(39.92 ± 19.12 in CKD; 45.95 ± 21.56 in HD; 47.13 ± 23.15 in KT; 51, 79 ± 18.89 in PD; p value = 0.321).” Specifically, this study confirms the importance of assessing post-transplant quality of life as it allows physicians to monitor the patient's self-perceived health and improve their adherence to treatment.

Gentile et al. (36) emphasized which variables may be needed to improve adherence to treatment such as: providing more detailed medical examinations, explaining immunosuppressive therapy and highlighting side effects, maintaining an adequate lifestyle, a healthy diet. The authors conducted a cross-sectional multicenter study with 1061 kidney transplant patients in France. For the evaluation of health-related quality of life (HRQOL), the “Short Form-36 health survey” (SF-36) and a questionnaire for “Recipient transplant quality of life” (RTQOL) were administered. The variance explained in the SF-36 regression models varies from 20 to 40% and from 9 to 33% for RTQOL. The study highlights how a careful evaluation of the socio-demographic variables (women, unemployment, lower education, life alone, high body mass index) and clinical aspects (diabetes, hypertension and hospitalization, a long duration of dialysis and side effects of treatment) are essential to optimize HRQOL.

Gheith et al. (37) focus on necessity of a good therapeutic adherence for the maintenance of the graft in the post-transplant. In particular, a questionnaire was administered, identified as a reliable method for the assessment of non-compliance, divided into two parts: the first part relating to demographic data and patient history, the second part relating to knowledge and awareness of the behavioral lifestyle recommended for subjects with kidney transplantation. Specifically, the second part included questions about: immunosuppressive therapy (dosages, regular intake, forgetfulness, side effects); nutrition (number of daily meals, presence of refined foods, etc., monitoring of body weight, control visits, infection prevention, physical activity, smoking, sun exposure, and sexual activity). From 22 to 48%, higher rates of non-compliance with immunosuppressive drugs have been reported. Any differences in compliance can be attributed to the length of the post-transplant time, age, gender and nature of the donor. A better adherence was found in subjects receiving the organ from a family donor (p value = 0.04). Analysis of the data showed that men were significantly more complier with drugs (p value = 0.02), while women were more compliant with recommended diets (p value = .03). In the case of infection prevention, the adhesion of the subjects to the measures to be adopted was partial (57%). Only 9% of patients avoid exposure to the sun. Regarding smoking, only 29.4% quit smoking after the transplant. Concerning sexual activity, 30% of subjects consulted the nephrologist for sexual problems. As far as physical activity are concerned, only 23% of the patients examined regularly practiced physical activity. In summary, kidney transplant patients examined in this study observe better adherence to drug therapy and less adherence to the suggested healthy lifestyle. Therefore the authors point out the need of a psychoeducational course of preparation for transplantation. Fundamental is the role assumed by the nurse who must provide patients undergoing a renal transplant the right knowledge of recommended lifestyle with the family support.

The study of Zelle et al. (57) analyzed the “fear of movement” related to daily “physical activity” (PA) in 487 renal transplant patients (RTR). The “Tampa Score of Kinesiophobia—Dutch Version” (TSK-11) was administered to evaluate the “fear of movement.” The Dutch translation of the “perceived physical activity scale” (LIVAS scale) was used to assess the perceived physical activity. Analysis of the data showed that subjects with a lower perception of their state of mental health had a greater “fear of movement” (p value = < 0.001), while the levels of physical self-efficacy were much lower (p value = < 0.001). The higher the scores on “fear of movement” in kidney transplanted subjects, the less physical activity was present daily (r = -0.22; p value < 0.001). The fear of movement was also associated with employment (r = -0.16; p value = 0.001), with sport (r = -0.12; p value = 0.01) and with free time PA (r = -0.12; p value = 0.02). This study demonstrates the importance of planning strategies to reduce fear of movement and optimize post-transplant physical activity for long-term maintenance of the graft.

Gordon et al. (39) observed the physical activity of kidney transplant patients, adherence to fluid intake recommendations and subjective behaviors related to psychophysical well-being. Semi-structured interviews on kidney transplant patients were carried out by telephone or in person within 2 months of transplantation. 90 out of 158 (57%) patients participated. The “Physical activity scale for the elderly” (PASE) questionnaire was used to assess physical activity. The PASE is a 21-element tool that evaluates physical activity in some areas: leisure sports, home activities, such as vacuum cleaners or lawn work and other activities, such as walking or using machinery in the last 7 days. Through the short form health survey (SF-36) the relationship between physical and mental health was assessed while the social support received, through the Medical Outcomes Study “Social Support Survey” (SSS). Analysis of the data showed that the majority of subjects (83%) were adherents regarding fluid intake, while half of the subjects (49%) reported high adherence to physical activity. It was shown that subjects of younger age and those with adequate social activities had greater self-efficacy with respect to physical exercise (r = 0.42; r = 0.30 p value < 0.01). The authors suggest that educational strategies should also focus on strengthening the sense of self-efficacy, thus stressing the importance of new information practices. This is particularly relevant as recent legislation states that “educational services” for subjects suffering from a renal disease are an indicator of improved quality of life.

#### Post-transplant Quality of Life Indicators

The in-depth analysis of various aspects of quality of life is necessary for monitoring transplanted patients and predicting results. The evaluation of the quality of life as an outcome measure of a solid organ transplant is an important tool, since it allows to highlight indicators of the general state of health useful for implementing any suitable treatments (medical, rehabilitation, psychological support) and getting the best from transplant surgery.

Kovacs et al. (42), through the cross-sectional observational study, state that kidney transplant patients (Tx) have a much better quality of life than patients on waited list (WL). Eight hundred eighty-eight transplant subjects and 187 subjects on the waiting list were studied. Quality of life studied with the Kidney Disease Quality of Life-Short Form (KDQoL-SF). The sub-scales evaluated were: “Physical functioning,” “General health perceptions,” “Energy/fatigue,” and “Emotional well-being.” Median scores were significantly higher for the Tx vs WL groups “(median physical function 80 vs 70 p value = 0.001; general perceptions on median health 50 vs 35 p value < 0.001; median energy/fatigue 70 vs 60 p value = < 0.001; emotional well-being median 80 versus 72, p value 0.003).” The symptoms of depression and the possible presence of sleep disorders were studied through the “Center for Epidemiologic Studies Depression” questionnaire (CES-D) and the “Athens Insomnia Scale” (AIS). Sleep disorders are less present in transplanted subjects compared to those on dialysis. Depressive symptoms are quite frequent in the subjects studied, but this symptomatology decreases after the kidney transplant (p value = 0.001). The authors suggest the importance of assessing quality of life after kidney transplantation.

Raiesifar et al. (49) conducted a three-month randomized clinical trial involving 90 kidney transplant patients divided into two groups: the treatment group included sessions to make the patient aware of the disease, even with the presence of family members, through lessons, informative and educational material on the transplant pathway, creating a need for follow-up. Instead, no specific intervention was performed in the control group. The “kidney transplant questionnaire” (KTQ-25), a designated tool for assessing quality of life in kidney transplant patients, was administered. The dimensions analyzed were: physical symptoms, fatigue, appearance, fear, and emotional insecurity. The scores on the questionnaire were high in the two groups of subjects, but the scores of the subjects belonging to the experimental group were considerably higher than those of the control group at 3 months. The mean total scores of KTQ-25 were 87.0 ± 6.2 (treatment group) and 88.0 ± 5.5 (control group) (p value < .001). This study underscores the importance of the continuing care model's effect on routine quality of life care among kidney transplant patients.

In a longitudinal study, von der Lippe et al. (54) evaluated changes in “health-related quality of life” (HRQOL) of subjects on dialysis compared to kidney transplant recipients in a cohort of 110 patients. HRQOL was observed through the “Kidney disease and quality of life-short form” (KDQOL-SF). General health improved after kidney transplantation (RTX), respectively from 58 ± 20 to 68 ± 21, p value < 0.001. In particular, it was found that with transplantation, younger subjects had better social relationships than older subjects (p value = 0.035). In general, the authors of the study showed an improvement in the quality of life in transplanted subjects compared to subjects on dialysis.

In a cross-sectional study of correlational design, Wei et al. (55) evaluated the “health-related quality of life” (HRQOL) of 88 kidney transplant (KT) subjects using the eighth SF-36 subscales: “Physical functioning” (PF), “physical role” (RP), “body pain” (BP), “vitality” (VT), “general perception of health” (GH), “social functioning” (SF), “emotional role” (RE), and “mental health” (MH). The results showed that with increasing age the scores in the subscales PF, RE, and BP decreased (r = -0.25, p value = 0.021; r = -0.21, p value = 0.047; r = -0.22, p value = 0.04, respectively). Scores on the PF subscale were lower in women than in men (76.2 versus 85.1, p value = 0.038). The subjects employed showed better scores at many subscales of the questionnaire compared to subjects not employed: PF (83.7 versus 73.8, p value = 0.034), RP (76.3 versus 52.4, p value = 0.013), GH (63.6 versus 51, 6, p value = 0.012), RE (78.3 versus 54.8, p value = 0.016), and MH (71.6 versus 63.3, p value = 0.039). The study reaffirms the importance of “health-related quality of life” (HRQOL) for a long-term compliance in kidney transplant subjects.

Shabany Hamedan and Mohamad Aliha (51) measured the relationship between adherence to immunosuppressive therapy and “quality of life” in 230 kidney transplant subjects. The “Immunosuppressant Therapy Adherence Scale” (ITAS) was used to assess adherence to immunosuppressive drugs. The questionnaire related to the study of quality of life has measured four dimensions: health/performance, socioeconomic, psychological/spiritual, family. The two questionnaires were completed by the patients. Evaluating adherence to immunosuppressive drug therapy, it was noted that adherence improved with older patients compared to younger ones [p value = 0.049, Chi-squared test (X²) = 8.873]; furthermore, it is negatively correlated with the time elapsed since transplantation (p value = 0.041, X² = 9.948) and with the amount of transplants performed (p value = 0.036, X² = 4.376). Data analysis pointed out that patients who adhered had a desirable quality of life: “health performance” [p value ≤0.0001 and correlation ratio eta (rETA) = 0.23], “socio-economic” (p value = 0.001 and rETA = 0.15), “psychological-spiritual” (p value = 0.011 and rETA = 0.15). Through this study the authors stressed that a good therapeutic adherence, should be an essential objective in the care of kidney transplant patients.

### Psychological Stability and Psychopathology

A total of 32 studies investigated psychological stability and psychopathology, 17 of them were high-quality studies and will be described.

#### Mental Distress and Psychological Symptoms

Kidney transplantation is an established treatment for end-stage kidney disease. However, it is a complex psychological experience that can generate mental distress and psychopathology. The affective profile in transplant patients should be examined in depth to highlight all the facets in their mental and emotional evaluation, which can represent easy barriers to treatment in post-transplantation.

De Pasquale et al. (9) explored behavioral and adaptation difficulties in kidney transplant patients through a graphical test: “Machover Draw-a-person test,” as the transplanted patient communicates with difficulty his deeper psychological contents. The variables emerging from the Machover Test regarded: “emotional coarctation” in 100% of the sample, “difficulty in interpersonal relationships and “anxiety” in 70% of the sample. The study showed that the design of the human figure (projective method) was useful for identifying and studying possible mental distress in post-transplant follow-up.

De Pasquale et al. (32) studied psychopathology, self-efficacy and quality of life in 120 kidney transplant subjects. The “Symptom Checklist 90 Revised” (SCL-90 R) was used to assess psychopathology. Self-efficacy has been studied with the “General self-efficacy scale,” quality of life with the “Short Form Health Survey” (SF-36). High self-efficacy was associated with a reduction in psychological symptoms “(self-efficacy/somatization, r = 0.145; self-efficacy/obsession, r = 0.557; self-efficacy/depression, r = 0.547; self-efficacy/Anxiety, r = 0.445; Self-efficacy/hostility, r = 0.528; self-efficacy/paranoia, r = 0.646; self-efficacy/psychosis, r = 0.264).” Self-efficacy is also positively correlated with a good quality of life on SF-36 “(Self-efficacy/Limitations of the physical role, r = 0.376; Self-efficacy/mental health, r = 0.493).” The authors pointed out that mental health and psychophysical well-being of these subjects was correlated with a good personal self-efficacy.

Mc Adams et al. (43) studied fragility and the quality of life subjectively perceived in 443 kidney transplant patients (2014–2017) using the “Kidney Disease Quality Of Life short form” (KDQOL-SF) instrument. Fragility was assessed through five components: “weight loss;” “weakness,” “exhaustion,” “low physical activity,” and “slowed walking speed.” Three months after transplantation, despite the reduced physiological reserve, frail subjects experienced an improvement in physical component of health related quality of life (HRQOL) (p value < 0.001) compared to non-fragile subjects. The study showed that even a high-risk group such as fragile kidney transplant (KT) recipients could experience the benefit of better HRQOL with KT.

Kofman et al. (41) in a retrospective multicenter study analyzed the presence of bipolar disorder (BD) and psychotic disorder in pre-transplantation and their evolution in 5 years post-transplantation. Forty-seven patients were identified including 34 with BD and 13 with psychotic disorder. During post-transplant follow-up half of the patients underwent various hospitalizations, within 60 months of the follow-up, due to psychiatric relapse [(incidence rate: 1.8/100 people-months; 95% confidence interval (CI); (1.2–2.7)] with poor therapeutic adherence to immunosuppressive drugs. This study suggested the need for psychiatric follow-up after transplantation in patients with pre-transplant psychiatric diagnosis.

#### Cognitive Impairments and Depressive Symptoms

The study of cognitive functions in kidney transplant patients has shown cognitive deficits, in particular impairments in verbal memory and executive functions, often associated with mood disorders. These adverse effects can persist and have a negative impact on all aspects of life. Post-transplant cognitive assessment can therefore be used to develop cognitive rehabilitation programs and other interventions in compromised transplant subjects.

Gelb et al. (35) compared the cognitive functions of42 kidney transplant patients with those of 49 healthy controls and 45 patients with chronic renalfailure. Verbal memory was studied through the “California Verbal Learning Test—SecondEdition (CVLT-II);” Executive functions were studied with the “Delis-Kaplan ExecutiveFunction System (D-KEFS).” Data analysis showed that subjects with chronic renal failure havecompromised cognitive functions than healthy controls [Color-word interference: Cohen’s d (d) = -0.68; learning and memory: d = -0.95]. The data is similar in transplant patients that have showed more compromised cognitive functions than healthy controls with low scores on Color-word interference (d = -0.56) and on the task of learning and memory (d = -0.74).

Goedendorp et al. (38) analyzed “severe fatigue” in 278 kidney transplant subjects, and the relationship between fatigue (concentration problems, reduced motivation and activity) and psychopathology related to kidney transplantation, such as depressive symptoms and sleep disturbances. “Severe fatigue” was studied using the “Checklist Individual Strength (CIS)—Subscale Fatigue.” Depressive symptoms were studied with “Beck Depression Inventory—Primary Care (BDI-PC).” Sleep disorders have been studied with the “Sickness Impact Profile 8 (SIP) —Sleep-Rest Subscale.” The study showed that 151 subjects showed a positive correlation between “depression, sleep disorders and fatigue (odd ratio = 9.70 and 1.02; p value = 0.013 and ≤0.001).”

Paterson et al. (45) analyzed the relationships between cognitive functions, depression, and adherence in 211 participants through a multivariate statistical analysis. Adherence was measured using the “Medication adherence self-efficacy scale-revised” (MASES-R); the measurement of cognitive functions included the use of an assessment comprising: the “Kaufman Brief Intelligence Test” (KBIT-2), the “California Verbal Learning Test” (CVLT-II) and the “Delis-Kaplan executive function system” (D-KEFS). Depression was assessed through the “Center for Epidemiological Studies Depression Scale” (CES-D); The authors suggested the influence of depressive symptoms and neurocognitive abilities in modifying therapeutic adherence [χ² (degree of freedom df = 313) = 376.24, p value=.009].

Troen et al. (53) evaluated the prevalence of cognitive dysfunctions and depression in transplant patients. The cognitive dysfunctions were evaluated with a battery of tests to measure: attention, memory, mental processing speed, and executive function. Depressive symptoms were studied through the “Center for Epidemiological Depression Scale” (CES-D) questionnaire. 30% of 183 participants had symptoms of mild to severe depression (score from 16 to 22 or higher). In the case of cognitive dysfunction, it was found that 33% had difficulty with memory tests, 58% showed deficits in attention tests and mental processing speed and 42% showed difficulties in executive tests. The study confirms the possible co-presence of mood and cognitive disorders in kidney transplant patients and reiterates the importance of monitoring cognitive and emotional aspects in the care of these patients.

The cross-sectional study by Weng et al. (56) analyzed clinical factors (anxiety and depression) and non-adherence variables in 252 subjects with renal transplantation for more than six months. The “Immunosuppressant Therapy Adherence Scale” (ITAS) was used to observe the adhesion variables. The evaluation of the symptoms of anxiety and depression was studied by the “Hospital Anxiety and Depression Scale” (HADS), while the “Interpersonal Support and Evaluation List-12” (ISEL-12) and the “Perceived stress scale—4” (PSS- 4) for the assessment of perceived interpersonal support and stress. The results of the study showed a significant correlation between non-adherence, depression (HADS p value= 0.02), perceived stress (PSS-4 p value = 0.04), lower family income (p value = 0.004), and lack of employment (p value = 0.03). The study suggests the need to plan interventions for the treatment of perceived stress, depressive symptoms and anxiety in order to increase adherence among kidney transplant recipients.

The study by Corruble et al. (30) showed a significant association between depressive symptoms reporting on the waiting list and post-transplant outcome. The “Short Form of Beck Depression Inventory” (Short-BDI) was used to assess depression. At baseline 51.6% of the evaluated subjects (n = 339) had depression “(Short-BDI score of 4 or more).” After 18 months from transplantation only 6.67% of patients (n = 6) had a graft failure among the 90 individuals (Short-BDI score higher than 7 at baseline). The study showed that depressive symptoms are not significantly associated with a negative transplant outcome.

Muller et al. (44) in a cross-sectional study studied the presence of anxiety and depression using the “Hospital anxiety and depression scale” (HADS-D/A) in kidney transplant patients. Regarding depression, the mean HADS-D scores were 4.48 ± 2.96; regarding anxiety the mean HADS-A scores were 5.01 ± 4.04. In particular, the authors pointed out that the prevalence of depression and anxiety remains surprisingly constant in patients after kidney transplantation.

#### Sleep Disorders, Anxiety, and Depression

Poor sleep quality is common after kidney transplantation and is one of the factors that adversely affects patients' quality of life after transplantation. This problem is associated with greater medical comorbidity and also with a state of emotional fragility. Therefore it is necessary to monitor changes in sleep quality and analyze the variables (anxiety, depression, fatigue, pain) that affect sleep quality in the first years after kidney transplantation, as these disorders represent important risk factors for non-adherence to treatments.

The study of Gross et al. (40) showed the presence of depressive symptoms (38%), anxiety (39%) and sleep disturbances (42%) in 138 transplant patients.

sThe “State-Trait Anxiety Inventory (STAI)” was used to assess anxiety, the “Center for Epidemiological Studies—Depression Scale (CES-D)” to assess depression and the “Pittsburgh Sleep Quality Index (PSQI)” for sleep disorders. Recipients of kidney, kidney/pancreas (n = 72) were included in a program based on stress reduction techniques, a training program for mindfulness meditation (awareness-based stress reduction—MBSR). The remaining patients (n = 66) were included in a health education program. After 8 weeks, in subjects who had undergone the “MBSR” program, anxiety, depression, and sleep disorders had declined, while quality of life had improved “(p value < 0.01, all)” and the benefits were maintained at 1 year “(p value < 0.05, all).” Instead, the results relating to the health education program were modest and not lasting. The study highlighted the importance and effectiveness of a non-pharmacological intervention for the treatment of post-transplant psychiatric symptoms.

Burkhalter et al. (28) studied sleep disorders in 249 kidney transplant subjects (RTx) transplanted into three Swiss transplant centers, through self-report questionnaires (Survey of Sleep—SOS) and more structured interviews. This study showed the high incidence of insomnia in kidney transplant patients (49.4% of the evaluated subjects). In 62.9% of the subjects sleep disorders were associated with the need to urinate. The authors of the study underline how important is the research in this field and the treatment of sleep disorders in subjects undergoing kidney transplantation.

Burkhalter et al. (29) evaluated the effect of “bright light therapy” (BLT) on sleep disorders and depression in adult patients with kidney transplantation (RTx) more than 1 year and diagnosed with sleep—waking disorders (SWD). This study included 30 subjects with RTx randomly assigned: BLT group or control group. Research has shown the presence of “chronic insomnia” in 42.5% of the examined sample. The “Depression, Anxiety and Self-reported Stress—21 elements” (DASS 21) was used to evaluate depression. The results showed that BLT improved significantly sleep timing. Moreover, depressive symptoms improved in BLT group “(basic intervention: 5.92–5.75 [standard error (SE): -0.28 (-0.87; 0.31)]” with progressive improvement up to follow-up: “5.75–4.08 (score > 5 means depressive symptomatology),” “[SE: -0.52 (-1.12; 0.08)].”

Pourfarziani et al. (47) evaluated sleep disorders in 39 kidney transplant patients using the “Pittsburg Sleep Quality Index” (PSQI) to evaluate the quality of sleep. The results of the study showed that 67% of patients were diagnosed as “poor sleepers” (PSQI total score > 5). The study confirms that sleep disorders are surprisingly common in renal transplant patients.

Reilly-Spong et al. (50) studied the prevalence, characteristics and correlates of sleep difficulties in 143 subjects who underwent solid organ transplantation; the recipients of the kidneys included about half of the sample (77 out of 143). Sleep quality was expected to have moderate to strong correlations with symptoms of depression, anxiety, fatigue and pain. Sleep disorders were measured with the “Pittsburgh Sleep Quality Index” (PSQI), Anxiety with the “State-Trait Anxiety Inventory—State Version” (STAI); depression was measured by the “Center for Epidemiological Studies—Depression Scale” (CES-D), fatigue (vitality) and pain with the “Subscale Short-Form 36 version 2” (SF-36). All transplanted subjects studied had sleep disturbances correlated (Pearson r) with depression (r = 0.61), anxiety (r = 0.58), fatigue (r = -0.58) and pain (r = -0.49) (p value < 0.001, all). The authors concluded that sleep disturbances are present in transplanted subjects and they are not adequately treated, therefore it is necessary to deepen them adequately and provide, for these subjects, educational strategies for a good quality of sleep.

The prospective study by Silva et al. (52) showed a high incidence of sleep disorders, with anxiety and depressive symptoms in 76 patients who underwent kidney transplantation, without significant changes in the two evaluation times (phase 1: 3-6 months; phase 2: 12 -15 months, after transplantation). Poor sleep prevalence, assessed with the “Pittsburg Sleep Quality Index” (PSQI) was slightly increased in the second evaluation time (36.7% in phase 1 and 38.3% in phase 2). The presence of anxiety (p value = 0.113) and symptoms of depression (p value = 0. 432), measured with the “Hospital Anxiety and Depression Scale” (HADS), were not significantly different at the two phases. The study confirms the presence of sleep disorders, anxiety and depression in the short and long term post-transplant period.

## Discussion

The data available to date suggest that pre and post- transplant screening process must include both a medical evaluation and a thorough psychological and psychiatric assessment for a better therapeutic adherence. Adherence is a very important issue in the medical care of kidney transplant patients (51). The life style and quality of life of a transplanted subject represent a key point, and it is essential to adhere to the therapy and prescribed behaviors (34, 79).

In the field of kidney transplantation, it is important to understand and be aware of the “seriousness” of the disease that caused the specific organ failure. However, it is necessary to realistically discuss what the transplantation means, to adequately inform the patient about transplant procedures and to support the willingness to face the surgery (10). Failure to adhere to immunosuppressive therapy may increase the risk of rejection of the transplanted organ and cause the loss of the latter. Non-adherence seems to be related to the complexity of medical prescriptions, the individual and clinical characteristics of the patient (personality traits, pre-transplant lifestyle including diet, physical activity, a long duration of dialysis) (36, 39, 46, 48). Moreover, it is important that the transplanted person accepts the support of their family and collaborates with the transplant team to adhere to the new lifestyle, such as the resumption of healthy exercise (37, 49, 57, 80). Also the return to work after a kidney transplant is an important indicator of psychophysical well-being (74, 83).

Organ transplantation is generally the last possible therapeutic act for chronic kidney disease and the process that precedes and follows its implementation subjects the patient to an important burden of psychological stress, with the possible appearance of psychopathology. The literature underline the need to evaluate, with adequate screening, the possible presence of psychopathology, such as anxiety, depression, cognitive impairment, sleep disorders in the kidney transplanted subjects, which can represent a negative factor for the good therapeutic adherence (42, 60, 72, 84).

Anxiety and depression are the most common disorders in kidney transplant recipients that may affect disease process and graft survival (12).

In the daily experience of transplanted patients, anxiety is a condition of alertness and apprehension that increases when infections occur, drug side effects and other conditions that expose the subject to a state of physical discomfort until the fear of rejection (44, 52).

Depressive episodes can manifest years after the transplant, when the subject has failed to plan adaptation coping strategies and he realizes that the transplant will not bring him back to the state of health that precedes kidney disease (38, 41, 71, 73).

In cases where depression reaches levels of clinical relevance, it would be good to treat it with antidepressant drugs and psychotherapeutic and supportive interventions and implement preventive education on patients and family members (40, 56).

Sleep disorders are very common in kidney transplant patients; the causes can regard demographic factors, lifestyles, disease-related factors, and social factors (28, 47, 52). If not adequately treated, they interfere with a good post-transplant quality of life and so they have recently stimulated interest of the researchers, with the need to investigate non-pharmacological interventions, such as “Bright light therapy” (BLT) (50, 58, 66, 69, 78).

Sleep disorders are less present in transplanted subjects compared to those on dialysis. Depressive symptoms are quite frequent both in transplanted and dialysis subjects, but this symptomatology decreases after the kidney transplant (42).

Often cognitive impairment is also found in kidney transplant patients. In particular those most analyzed in the reported studies concern: attention, visual spatial memory, working memory and problem solving (35, 53). It has been shown that cognitive disorders in kidney transplant recipients can influence therapeutic adherence (45, 85).

If the risk factors that could influence the correct approach to the therapeutic scheme are identified, it will be possible to optimize the patient's resources and at the same time establish a good therapeutic adherence (16, 33, 51, 56). In fact, following the transplantation, it is necessary that the patient actively participates in his own care pathway, through the regular intake of drugs, the correct maintenance of follow-up and the maintenance of a lifestyle in accordance with medical indications in order to allow him a satisfactory quality of life. It is essential that kidney transplanted subject be aware of the best quality of life (psychological well-being, general health, vitality) found after the transplant compared to the time of dialysis. It may happen that lack of awareness of the “change” and living a poorly recommended lifestyle endanger the survival of the graft (34, 54).

Psychiatric and psychological support is often necessary to favor the process of adaptation to transplant in the long term, since aspects of psychopathology have been analyzed by different authors in the post-transplantation, with results of lower levels of adherence (59, 61, 63, 65).

Future directions of research should focus on deepening all those factors (self-efficacy, awareness disease, social and family support, health education) that influence the transplant preparation pathway and post-transplant results (32, 80). Furthermore, the development of interdisciplinary interventions with the various specialists (surgeon, nephrologist, psychiatrist and psychologist) would be desirable to identify shared operational protocols for integrated care of the transplanted patient and also plan training and updating courses for the “health team” with particular attention to psychological, communicative and relational aspects (24, 62, 64, 67, 68, 70, 75–77).

## Data Availability Statement

The datasets generated for this study are available on request to the corresponding author.

## Author Contributions

CP, MP, VM, and MV contributed conception and design of the study. They participated in drafting the work. PZ, NB, GB, LI, AG, and PV have made substantial contributions to the conception of the work and interpretation of data. They participated in revising the work critically for important intellectual content. All authors contributed to manuscript revision, read and approved the submitted version.

## Conflict of Interest

The authors declare that the research was conducted in the absence of any commercial or financial relationships that could be construed as a potential conflict of interest.
